# Residual bone mass after TLIF treated by TELD under local anesthesia: a case report

**DOI:** 10.3389/fsurg.2026.1874018

**Published:** 2026-07-29

**Authors:** Haining Zhou, Zefang Li, Zenghui Zhao, Xiaoji Luo, Ningdao Li

**Affiliations:** 1 Department of Orthopaedic Surgery, Chongqing Municipal Health Commission Key Laboratory of Musculoskeletal Regeneration and Translational Medicine/Orthopaedic Research Laboratory, The First Affiliated Hospital of Chongqing Medical University, Chongqing, China; 2 Department of Orthopaedic Surgery,Chongqing University Qianjiang Hospital, Chongqing, China; 3 Department of Orthopaedic, Chongqing General, Hospital Chongqing University, Chongqing, China; 4 Department of Orthopedics, The First Affiliated Hospital of Chongqing Medical and Pharmaceutical College, Chongqing, China

**Keywords:** nerve root compression, residual bone fragment, revision endoscopic surgery, TELD under local anesthesia, transforaminal lumbar interbody fusion

## Abstract

**Purpose:**

This paper introduces a postoperative revision technique for transforaminal lumbar interbody fusion, namely, removing a free bone fragment under TELD (transforaminal endoscopic lumbar discectomy) under local anesthesia. It is used to treat the corresponding symptoms caused by the residual bone mass continuously compressing the nerve after transforaminal lumbar interbody fusion.

**Methods:**

Patient with a residual bone fragment after lumbar interbody fusion was treated by removing a free bone fragment under TELD under local anesthesia.

**Results:**

A case of a patient who underwent transforaminal lumbar interbody fusion (TLIF) and had a free bone fragment compressing the nerve roots after the operation was successfully treated by removing the free bone fragment under TELD under local anesthesia. After the removal, the patient's symptoms in the right lower limb improved significantly, and the pain and numbness were greatly reduced. During the subsequent four-month follow-up period, the symptoms continued to ease, and there were no other complications.

**Conclusion:**

The free bone fragment removal under TELD under local anesthesia used in this study is safe and effective, and it can be applied to the treatment of nerve compression caused by the free bone fragment after transforaminal lumbar interbody fusion. We have adopted the AOSpine abbreviation TELD (transforaminal endoscopic lumbar discectomy) and assigned the case to complexity grade IIb (prior surgery + foraminal pathology) per the 2025 two-dimensional classification.

## Introduction

Transforaminal lumbar interbody fusion is a common open surgery for treating lumbar intervertebral disc protrusion accompanied by sciatica. Its advantages lie in directly decompressing the intervertebral disc to relieve the compression on the nerve roots, completely removing the intervertebral disc, using internal fixation to stabilize the spine, and maximally reconstructing lumbar function (1, 2). This surgical approach yields favorable surgical outcomes for patients with multi-segment intervertebral disc protrusion or those accompanied by lumbar spondylolisthesis (3). However, precisely because it is an open surgery, the trauma is relatively significant. Unknown situations can occur at any time during the operation, and there are numerous postoperative complications (4). Postoperative residual bone mass compressing nerve roots is a relatively rare complication, but it has a certain impact on the postoperative recovery of patients. When the residual bone from bone grafts or laminectomy produced during the operation enters the spinal canal and compresses nerves, new radiculogenic symptoms may occur, which require intervention, whether through open surgery or minimally invasive surgery (5). However, the postoperative revision of traditional lumbar interbody fusion typically necessitates extensive muscle dissection, scar tissue excision, and even the removal of internal fixation devices to attain the revision objective. In comparison with minimally invasive revision surgery, it is riskier, more invasive, inflicts more pain on patients, and there are risks such as nerve damage, infection, and failure of re-fusion after the operation (6). In contrast, with the maturation of interlaminar endoscopy technology, many issues that previously required open surgery can now be addressed using this technique. Even procedures such as the removal of a single herniated disc or the excision of an intraspinal tumor can be performed with the assistance of percutaneous endoscopic discectomy (7–9). TELD technology for spinal surgery offers distinct advantages, including faster postoperative recovery, reduced blood loss, and fewer complications compared to open surgery. Therefore, this report provides a detailed account of a successful case of innovative treatment of a residual bone fragment after transforaminal lumbar interbody fusion using an TELD under local anesthesia.

## Case report

**Figure 1:**
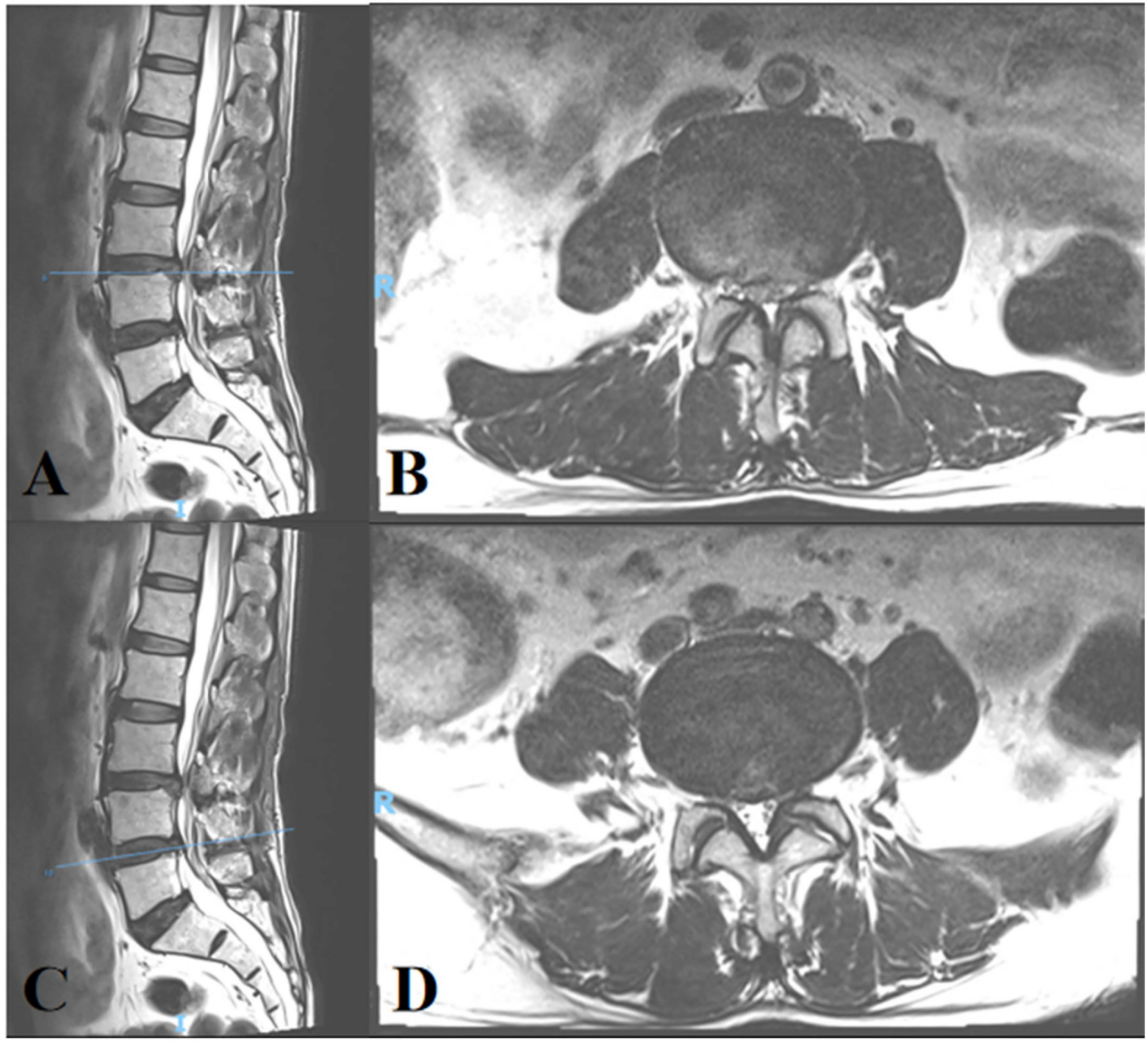
Magnetic resonance imaging (MRI) shows that both L3–4 and L4–5 have lumbar intervertebral disc protrusion. L3–4 sagittal view; axial view of L3–4; L4–5 sagittal view; L4–5 axial view.

A 66-year-old male presented for medical treatment due to low back pain lasting over 20 years, which had worsened, along with pain in both lower extremities, for 6 years. The main symptoms are low back pain, paroxysmal pain on the anterior and lateral sides of the lower legs, accompanied by a feeling of soreness, distension, and numbness. The symptoms on the right side are more severe and worsen after prolonged sitting, standing, or walking. The patient went to a local hospital for physical therapy on his own, but saw no significant improvement. So they came to our outpatient department for further diagnosis and treatment. Lumbar MRI examination revealed intervertebral disc protrusion at L3/4 and L4/5 (Figure 1). The patient's surgical indications are clear. Considering the patient's multi-level protrusion (L3/4 and L4/5 intervertebral disc protrusion), after discussing the surgical plan and related risks with the family and the patient himself, it was proposed that transforaminal lumbar interbody fusion + lumbar nerve root decompression be performed.

After general anesthesia, pedicle screws were implanted bilaterally at L3–L5. Preoperative MRI revealed severe stenosis at L3/4, We measured the anteroposterior diameter of the L4/L5 spinal canal using preoperative axial MRI, which was 12.88 mm (consistent with the normal anteroposterior diameter of the lumbar spine ≥12 mm). and the lateral-recess width was 1.96 mm (normal >3 mm), Therefore, this patient does not exhibit lumbar spinal canal stenosis at the L4/L5 level but demonstrates lateral recess stenosis. Since the symptoms observed on the lateral aspect of the lower leg were consistent with manifestations of L5 nerve root compression, the L4/L5 segment also required treatment. After endoscopic exploration, the L4/5 annulus was found intact and the nerve roots were freely mobile; therefore, we elected to perform decompression and instrumented fusion only at L3/4, with L4/5 screws placed prophylactically for stability, but without interbody fusion. Then, we performed extensive bone decompression on L3–L4, including resecting the bilateral L3-inferior articular processes, the lower edge of the L3 lamina, the upper edge of the L4 lamina, and the medial edge of the L4-superior articular processes (with obvious hyperplasia and significant stenosis of the lateral recess). Then, the thickened ligamentum flavum of L3–L4 was removed. Under 3D endoscopy, the intervertebral disc was resected, and the L3/L4 nerve roots were released. After dilating the intervertebral space and scraping the endplate, the autologous bone and PEEK fusion device were implanted. The graft volume is approximately 5 g, the composition is morselized autologous bone from the resected laminae and articular processes, and then implanted graft into the anterior in the disc space. We place the drainage tube, then fix the titanium rod and close the wound.

**Figure 2:**
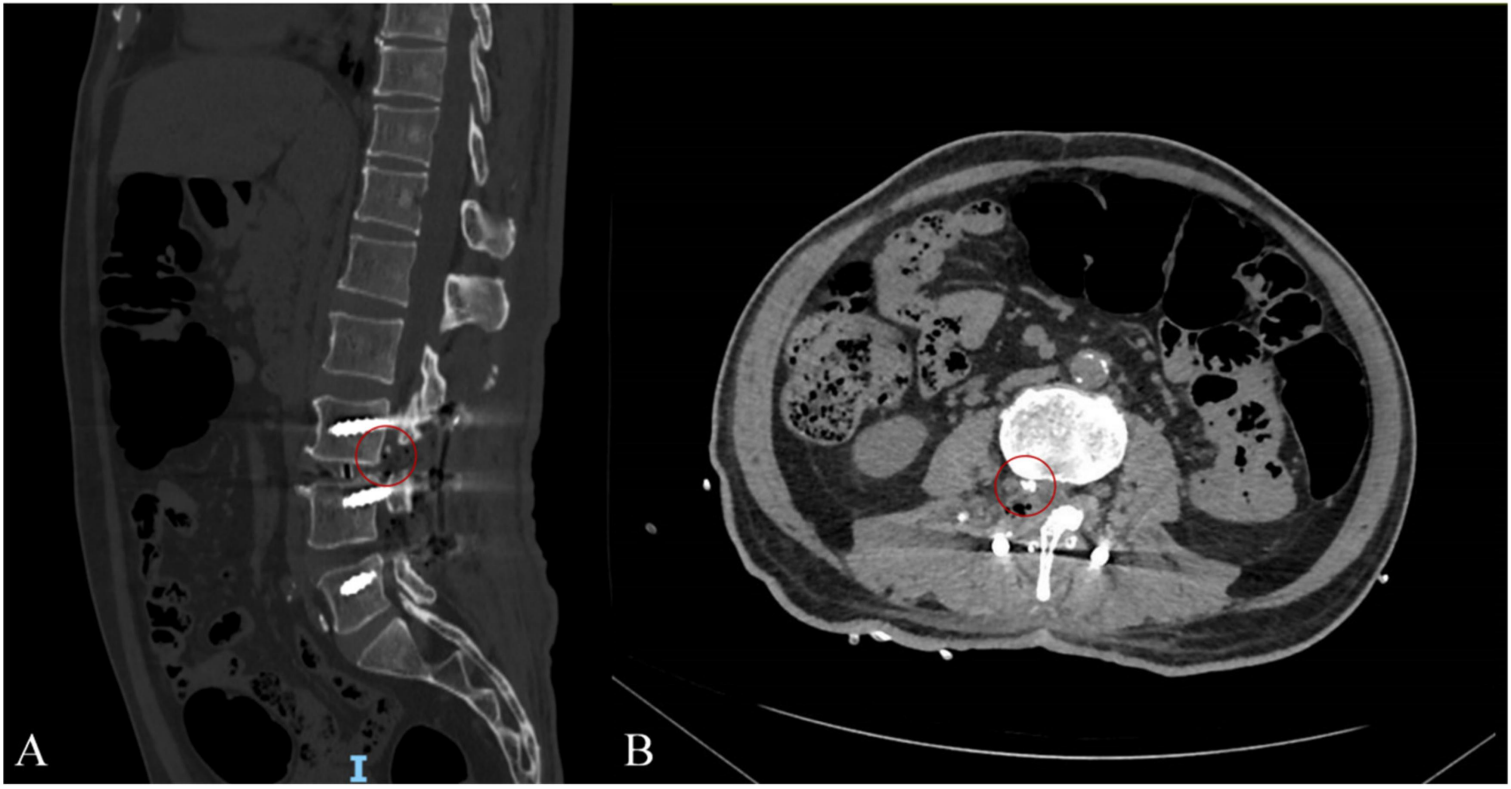
**(A)** Shows the lumbar CT on the sagittal view indicating residual bone mass continuously compressing the nerve roots, while **(B)** is its axial view.

The patient safely returned to the ward after the operation. After one day of observation, the patient reported that the lower back pain had been significantly relieved compared to before, and the pain and numbness on the anterolateral side of the lower legs had been significantly reduced. However, three days later, the pain and numbness on the anterior side of the right thigh continued to worsen, and We administered intravenous infusion of a mixture containing 0.5 mg of flurbiprofen ester injection and 100 mg of 0.9% normal saline solution for pain relief, but it failed to effectively alleviate the symptoms. We immediately completed the lumbar CT (Figures 2A,B), which indicated that there was a residual bone fragment under the axilla of the right L3 nerve root, and it was continuously causing compression. It was considered that there was compression of a bone-graft particle on the right L3 nerve root, which resulted in the corresponding symptoms of the L3 nerve root. At this time, the patient had indications for a second operation. Similarly, prior to the surgery, we discussed and ruled out other potential complications, the most typical of which was those caused by screw implantation. However, the lumbar CT (Figures 2A,B) confirmed that the right L3 pedicle screw was placed in an acceptable position (Gertzbein-Robbins grade A), with no breach of the medial or inferior wall, thus we excluded screw-related radiculopathy. The patient is generally in poor condition (Grade 3 hypertension is at very high risk; there is a history of diabetes and coronary heart disease. There is right hemiplegia). Because he has a history of diabetes, postoperative wounds may be difficult to heal, and infections may also occur. If minimally invasive surgery was chosen, we had the option between posterior routes (e.g., biportal/interlaminar endoscopy) and the transforaminal approach; we were inclined to the transforaminal approach, because the residual bone fragment was located at the axilla of the right L3 nerve root. The posterior route, though previously opened, was heavily obstructed by the existing pedicle screws; the transforaminal route allowed direct access to the fragment without violating the posterior elements. Also, the lamina was resected, making local anesthesia particularly challenging and increasing the risk of nerve root and dural sac injury. After thorough discussion, communication with the patient and their family about a second operation, and multi-disciplinary consultation and assessment, taking into account the patient's overall poor condition, five days after the first fusion surgery, it was proposed to perform an TELD residual bone mass removal under local anesthesia.

## Technique description

### Patient positioning, OR setup, and under local anesthesia

The patient was placed in a prone position, with pillows placed on the chest and iliac region to suspend the chest and abdomen. The C-arm x-ray fluoroscopy was used to locate and mark the L3/4 space. Routine disinfection was performed, and sterile wipes were laid out. The anesthesia protocol was pure local infiltration anesthesia: a mixture of 5 mL of 1% lidocaine, 2.5 mL of 0.75% ropivacaine, and 10 mL of normal saline was injected layer by layer ((skin, subcutaneous tissue, deep fascia, and finally, we Injected anesthetic around the intervertebral foramen to allow its diffusion.) by the surgeon. Vital signs (heart rate, blood pressure, and SpO_2_) were continuously monitored by the anesthesia doctor and remained stable within normal limits.

### Surgical approach

**Figure 3:**
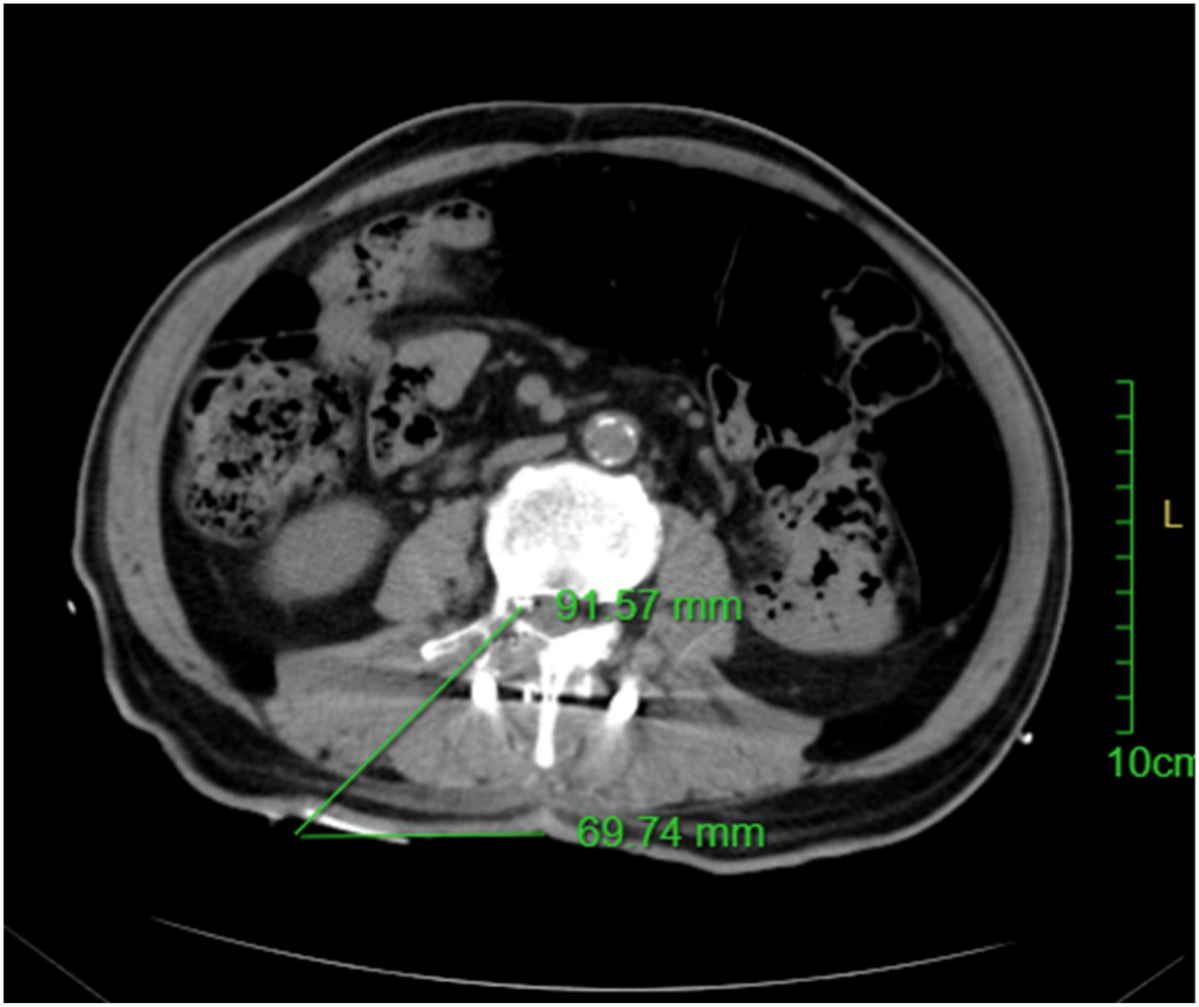
The puncture point, the trajectory of the working channel, and the spatial relationship with the bone block are marked. It can be seen that a distance about 7 cm from the patient's midline is the most appropriate point for needle insertion.

A SPINENDOS® full-endoscopic system (Model SP081375.030) was used for the procedure, with the following specifications: outer diameter 6.3 mm, working channel inner diameter 3.75 mm, working length 181 mm, 30° oblique viewing angle, and 80° field of view. A technical diagram illustrating the preoperative measurement and puncture trajectory is provided in Figure 3. We take 7.0 cm away from the midline of the patient as the puncture point. Under x-ray guidance, a puncture guide rod was placed on the ventral side of the upper articular process on the right side of the L3/4 intervertebral foramen. Then we inserted the working sleeve and removed the puncture guide rod. After doing all this, we inserted the intervertebral foramen endoscope.

### Endoscopic neural decompression

**Figure 4:**
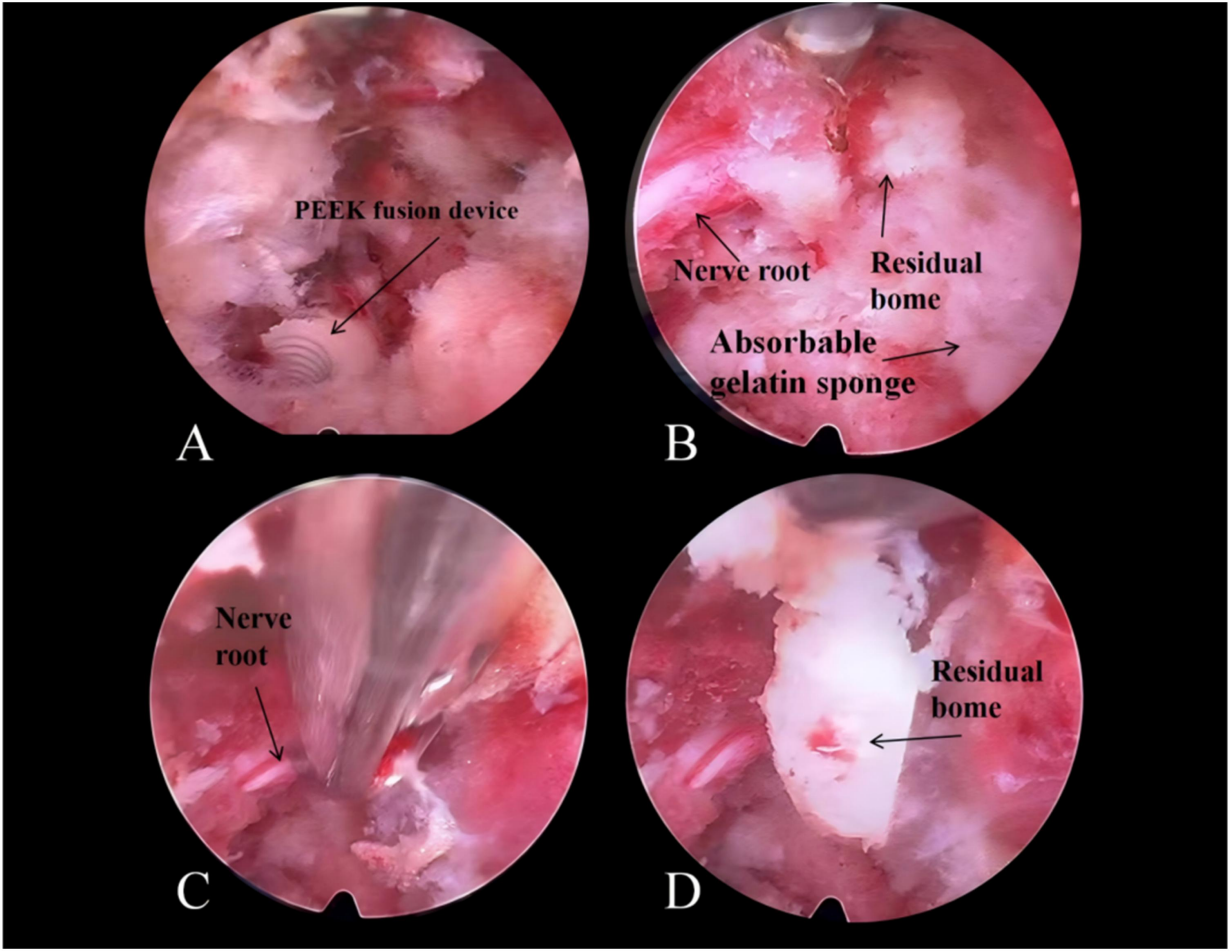
In figure **(A)**, it can be seen that the nerve root is compressed by a grayish-white bone block. **(B)** Shows the removal of the surrounding soft tissue using a nucleus pulposus forceps. The absorbable gelatin sponge is from the initial TLIF surgery. **(C)** Shows the release of the L3 nerve root; **(D)** shows the careful removal of the pressing bone mass.

We carefully searched for and confirmed the compressed right L3 nerve root under endoscopy. It was evident that the nerve root was being compressed by a grayish-white, hard bone fragment (Figure 4A). We carefully separated the adhesions between the bone fragment and the nerve root, as well as those with the surrounding soft tissue, using a nerve stripping tool (Figure 4B). We then carefully released the compressed nerve roots again (Figure 4C). After the bone fragment was fully free, we completely removed it by clamping with a nucleus pulposus forceps (Figure 4D). Due to the small size of the bone, with the help of an endoscope, we removed the compressed bone under direct vision without any fragments in the process to prevent small fragments from re-migrating into the spinal canal. Bipolar radiofrequency ablation was used to stop the bleeding. This kind of movement requires a careful dissection by an experienced surgeon to avoid nerve damage. A C-arm x-ray fluoroscopy was performed again to ensure that the bone mass was completely removed. Owing to the advantages of the local anesthesia. We can communicated with the patient at any time during the procedure. Consequently, after the removal of residual bone fragments, we immediately received positive feedback from the patient, confirming that the pain and numbness in the right thigh had been alleviated.

### Closure

After careful examination under the intervertebral foramen endoscope and confirmation that there are no bleeding points within the field of view, the incision was closed with a single 3-0 absorbable suture.

### Outcomes

**Figure 5:**
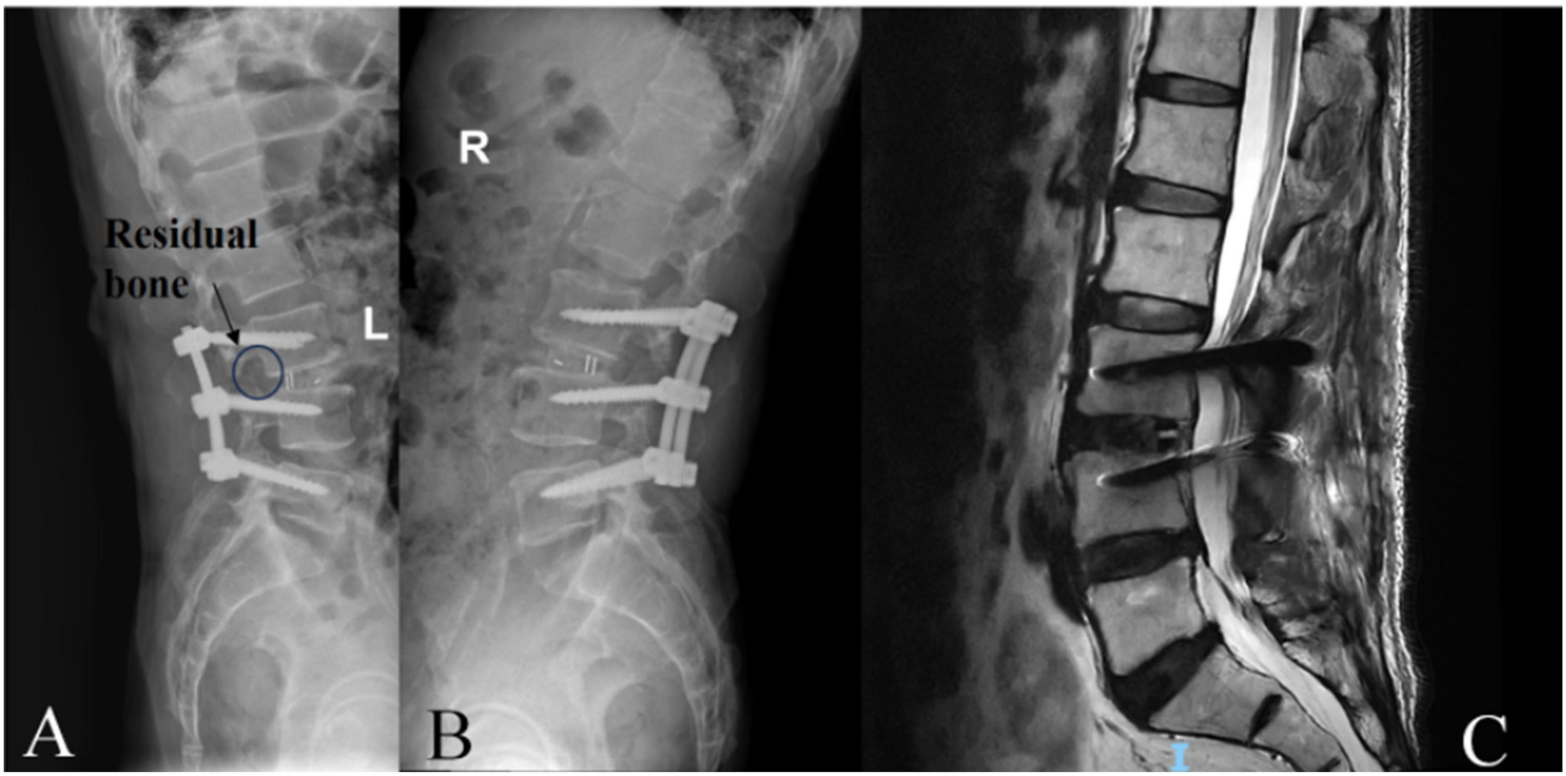
In the circle marked in figure **(A)** (preoperative before revision), there is a bone mass continuously compressing the nerve. In figures **(B**,**C)** (postoperative 4-month), at the same position, no bone fragments can be seen remaining at all

The operation was completed smoothly, and the patient was safely returned to the ward. The operation lasted for 55 min, and there was 5 mL of blood loss during the operation. During this period, the patient's vital signs were stable. When the patient returned to the ward, mannitol was administered to alleviate nerve root edema, and flurbiprofen axetil injection was used to relieve postoperative pain. The patient was discharged on the third day after the operation. During the follow-up period (4 months), the patient reported significant relief of low back pain and symptoms in both lower extremities, Especially, the pain on the front side of the right thigh was significantly improved before the revision surgery, with no other discomforts. The clinical outcomes before and after the revision procedure aresummarized in Table 1. Four months after the operation, the patient's re-examination of the lateral x-ray and MRI of the lumbar spine indicated that the bone mass had been completely removed (Figure 5).

**Table 1:** Clinical outcomes and neurological examination before and after revision surgery.

Time point	VAS (back)	VAS (right thigh)	Right L3 motor strength	Right thigh anterior sensation	SLR test
Pre-revision	2	7	4/5	Marked hypoesthesia	60° (with radiating pain)
Immediate post-revision	1	2	5/5	Mild hypoesthesia	70° (slight discomfort)
Discharge (day 3)	1	1	5/5	Slightly reduced	75° (negative)
4-month follow-up	0	0	5/5	Near-normal	80° (negative)

VAS, Visual Analogue Scale (0–10); SLR, straight leg raise. The patient had a pre-existing history of right hemiplegia due to prior stroke; the motor strength improvement reflects relief of nerve root compression rather than recovery from central causes. ODI was not assessed at the time of this retrospective report; we will include it in the ongoing long-term follow-up.

## Discussion

In this case, following the transforaminal lumbar interbody fusion surgery, the compression on the L4 and L5 nerve roots was alleviated. However, the remaining bone mass compressed other nerve roots (specifically, the right L3 nerve root), resulting in persistent pain at a different site compared to that before the surgery. By utilizing the endoscopic technique of the intervertebral foramen under local anesthesia, precise removal was accomplished. This case is highly beneficial for the clinical treatment of similar cases.

Transforaminal lumbar interbody fusion (TLIF) has always been a common surgical method for treating lumbar intervertebral disc protrusion (10). Compression of non-target nerve roots by residual bone fragments is a relatively uncommon complication that potentially results from migrated bone graft particles. In this case, the complication of residual bone fragments compressing the nerve may occur due to repeated compression of the gelatin sponge during the first surgical procedure for hemostasis, which displaced the implanted autologous bone graft into the L3 nerve root sheath and secured it firmly. We admit that this is a reasonable guess based on a postoperative review of the entire first surgery.

The migration of residual bone fragments or bone graft material into the spinal canal, epidural space, or even the intradural space following lumbar spinal fusion is a rare but serious complication, and similar cases have been reported in the literature. Gu et al. conducted a retrospective analysis of 23 such cases and found that the most common source of ectopic bone fragments was loss of intervertebral fusion graft material or residual material from fenestrated fusion (14/23 cases); All patients developed radiating pain in the limb on the decompressed side or the contralateral side postoperatively, with onset ranging from immediately after surgery to 2 weeks postoperatively (mean 3.2 ± 1.7 days). In managing this complication, open revision and endoscopic revision each have their own characteristics. Open revision surgery has long been the primary method for addressing such complications. Among the 23 cases reported by Gu et al., 3 patients underwent emergency posterior open nerve root exploration and bone removal (performed either intraoperatively or on the day after surgery), and 5 patients underwent posterior open surgery for bone removal following failure of conservative treatment; postoperative pain symptoms were relieved in all patients (5). In the case of intradural bone migration reported by Xie et al., the bone fragment was also successfully removed via emergency revision surgery (11). In the cauda equina syndrome case reported by Yang et al., emergency dural lysis was performed to completely remove the intradural bone graft material (12). However, open revision surgery typically requires extensive muscle dissection and resection of scar tissue; against the backdrop of altered anatomical structures and scar adhesions following the initial fusion procedure, the risks of nerve injury, infection, and failed re-fusion are significantly increased. For elderly patients with multiple underlying conditions, the surgical risks of open revision are particularly pronounced. Spinal endoscopic revision surgery, as a minimally invasive alternative, has gradually gained attention in recent years. In a series by Gu et al. (5), two cases successfully underwent nerve root exploration and bone fragment removal using spinal endoscopy. Slagel et al. reported a case of allograft migration following MIS-TLIF surgery, in which the graft was also removed via a second procedure (13). Compared to open revision surgery, endoscopic revision offers the advantages of minimal trauma, reduced blood loss, and faster postoperative recovery. Furthermore, it can be performed under local anesthesia, allowing for real-time patient feedback during the procedure, thereby reducing the risk of nerve injury. Also, the partial removal of the articular processes during the initial surgery provides a natural operative pathway for endoscopy. In summary, open revision surgery is suitable for critically ill patients with deep-seated bone fragments, accompanied by dural injury or cauda equina syndrome; endoscopic revision, on the other hand, is more suitable for cases where the bone fragment's location is clearly defined, situated in the intervertebral foramen or paravertebral recess, and where the patient has multiple underlying medical conditions. Against this backdrop, this case report demonstrates the successful application of endoscopic removal of residual bone fragments through the intervertebral foramen under local anesthesia in high-risk patients, providing a new minimally invasive option for the treatment of this complication. On the other hand, the descriptions in the aforementioned literature also indirectly indicate that this complication is relatively rare. Hunt et al. reported an incidence of 1.9%–5.9%, and Gu et al. identified only 23 cases over seven years. Xie et al. and Slagel et al. also described it as a “rare” complication, underscoring its low prevalence.

Although autograft in lumbar spinal fusion achieves fusion rates of up to 70% and yields good clinical outcomes in 92% of patients (10), it carries the risk of this complication (5). When it occurs and causes symptoms like pain and numbness, patient satisfaction and overall outcomes are negatively impacted (14), potentially prolonging hospitalization and increasing costs. This complex scenario presents a critical challenge: how to precisely remove the compressing fragment while minimizing the morbidity of revision surgery.

This report addresses this challenge. Spinal surgery is highly complex and often requires thorough decompression of the nerve roots. Therefore, when nerve roots or the dural sac are compressed or damaged, it often leads to new symptoms. There was once a report introducing the removal of a broken pedicle guide wire during a posterior lumbar interbody fusion surgery under foraminal endoscopy (15). Numerous cases have demonstrated the successful retrieval of surgical blades left behind during spinal procedures using transforaminal endoscopy (16, 17). There have also been cases in which residual lumbar drainage tubes were removed via transforaminal endoscopy (18). However, there is no report describing the use of TELD under local anesthesia in high-risk patients to alleviate symptoms caused by residual bone fragments compressing nerves after lumbar fusion surgery. Based on the clinical experience and summaries of surgeons, as well as previous similar reports, there are two mainstream treatment options when conservative treatment is ineffective. First, open surgery was conducted to explore the bone fragment. Second, the bone fragment was explored and separated under the intervertebral foramen endoscope. For patients with clear preoperative localization and significant comorbidities, endoscopic removal offers distinct advantages. Often, the facet joints have already been resected during the initial TLIF, which facilitates endoscopic access.

Applying transforaminal endoscopy for residual fragment removal represents an innovative and valuable technical advancement. Firstly, thorough preoperative imaging (CT/x-ray) is crucial for localizing the fragment, correlating it with symptoms, and guiding the endoscopic approach. In this case, the patient did not experience postoperative complications such as wound infection or symptom aggravation after the operation. The incision for the second operation was only 7 mm, and the intraoperative blood loss was only 5 mL. The patient was discharged 3 days after the operation, which significantly shortened the recovery period. Moreover, there was no recurrence during the 4-month follow-up after the operation. Secondly, all the instruments we used during the operation were conventional ones for TELD discectomy, and there was no need to purchase or design new surgical tools. In addition, the intervertebral foramen endoscope could directly reach the position where the bone mass was causing compression. Combined with the wide field of vision under the endoscope, the bone mass could be precisely removed. Moreover, under local anesthesia, we were able to continuously monitor the patient's pain levels throughout the procedure, we have a continuous verbal communication with the patient to confirm consciousness and identify the source of pain (whether it is surgical-induced low back pain, intraoperative injury, or radiating pain caused by nerve traction). During the procedure, we verified the adequacy of neural decompression through real-time patient feedback. Specifically, when the bone fragment was gently dissected and removed, we immediately confirmed with the patient whether the right anterior thigh pain was alleviated. This real-time verbal feedback served as a critical safeguard and confirmed successful decompression intraoperatively.

The key points and difficulties of the surgery lie in: 1) Precise positioning: By combining preoperative X-rays and CT scans, the approximate position of the bone block was identified. During the operation, the C-arm was used to precisely locate the target laminar space and puncture point, thereby establishing the surgical access. 2) Residual bone fragment identification and treatment: The bone block was located relatively deep. During the operation, the surgeon carefully explored the nerve roots to avoid forced pulling that could cause nerve damage. Then, they used a nucleus pulposus forceps to ensure that the bone block was completely removed to prevent fragmentation and residue. This transforaminal endoscopic(TELD) removal under local anesthesia is particularly suitable for elderly patients with significant comorbidities, when the residual fragment is well-localized within the foramen or lateral recess, and when open revision carries high risk.

This study, as the first technical report on “removing bone blocks that have compressed nerve roots through secondary surgery”, also has many inherent limitations: 1) The evidence level of a single case is limited. Moreover, the follow-up period for this patient was four months, and there is a lack of long-term follow-up data. We are continuing the follow-up and plan to report the 2-year outcomes, especially regarding the unfused L4/5 segment. 2) Technical success heavily depends on the surgeon's experience and skill. 3) The position of the bone block also determines the feasibility of the surgery. When the bone block is in the high segment, it is more likely to stimulate the nerve outlet roots, and the TELD technique is relatively more challenging.

Also, as a reflection on the occurrence of this complication, we acknowledge that a two-level fusion might have contained the graft material and potentially prevented this complication. However, at the time of surgery, the patient's comorbidities and relatively mild pathology at L4/5 led us to choose a single-level fusion. In retrospect, when residual bone fragments are at risk, additional containment measures (e.g., using a cage with a cover or a barrier) should be considered.

## Conclusion

We introduce an innovative application of surgical methods, namely, the removal of the bone mass causing nerve compression under TELD under local anesthesia. This technique provides surgeons with a new solution for handling residual bone fragments after lumbar interbody fusion surgery.

## Data Availability

The original contributions presented in the study are included in the article/Supplementary Material, further inquiries can be directed to the corresponding author.
